# Asymmetric dimethylarginine attenuates serum starvation-induced apoptosis via suppression of the Fas (APO-1/CD95)/JNK (SAPK) pathway

**DOI:** 10.1038/cddis.2013.345

**Published:** 2013-10-03

**Authors:** H Li, Y Zhou, A Zhao, Y Qiu, G Xie, Q Jiang, X Zheng, W Zhong, X Sun, Z Zhou, W Jia

**Affiliations:** 1 Center for Translational Medicine, Shanghai Jiao Tong University, Affiliated Sixth People's Hospital, Shanghai 200233, China; 2 Center for Translational Biomedical Research, University of North Carolina at Greensboro, North Carolina Research Campus, Kannapolis, NC 28081, USA; 3 David H Murdock Research Institute, North Carolina Research Campus, Kannapolis, NC 28081, USA; 4 University of Hawaii Cancer Center, Honolulu, HI 96813, USA

**Keywords:** ADMA, colon cancer, apoptosis, Fas, JNK, chemotherapy

## Abstract

Asymmetric dimethylarginine (ADMA) is synthesized by protein arginine methyltransferases during methylation of protein arginine residues and released into blood upon proteolysis. Higher concentrations of ADMA in blood have been observed in patients with metabolic diseases and certain cancers. However, the role of ADMA in colon cancer has not been well investigated. ADMA serum levels in human patients diagnosed with colon cancer were found to be higher than those present in healthy subjects. ADMA treatment of LoVo cells, a human colon adenocarcinoma cell line, attenuated serum starvation-induced apoptosis and suppressed the activation of the Fas (APO-1/CD95)/JNK (SAPK) (c-Jun N terminal protein kinase/stress-activated protein kinase)pathway. ADMA also suppressed the activation of JNK triggered by death receptor ligand anti-Fas mAb and exogenous C_2_-ceramide. Moreover, we demonstrated that ADMA pretreatment protected LoVo cells from doxorubicin hydrochloride-induced cell death and activation of the Fas/JNK pathway. In summary, our results suggest that the elevated ADMA in colon cancer patients may contribute to the blocking of apoptosis of cancer cells in response to stress and chemotherapy.

Asymmetric dimethylarginine (ADMA) is an endogenous metabolite present in blood, tissues, and cells. ADMA is produced upon methylation of arginine residues in proteins by protein arginine methyltransferases, and liberated upon hydrolysis of methylated proteins.^1^ ADMA is a competitive inhibitor of nitric oxide synthase,^2^ and thereby plays an important role in cardiovascular biology.^3, 4, 5^ Increased levels of ADMA have been observed in a variety of diseases, including hypertension,^6^ atherosclerosis,^7^ diabetes,^8^ heart failure,^9, 10^ and pre-eclampsia in pregnant women.^11^

A global gene expression analysis in human coronary artery endothelial cells treated with 100 *μ*M ADMA showed that 86 genes were significantly altered (*P<*0.05, fold change >1.7). Many of the altered genes are involved in the regulation of cell proliferation, cell cycle, and RNA splicing processes.^12^ Recent studies show that plasma ADMA levels are higher in several types of cancers, such as lung cancer, hematopoietic tumor, gastric cancer, and breast cancer,^13, 14^ suggesting that ADMA may also be involved in tumor development. ADMA is mainly generated via a metabolic process regulated by dimethylarginine dimethylaminohydrolase (DDAH) and enhanced expression of DDAH1 has been reported to promote tumor growth *in vivo*, accompanied with decreased ADMA levels and increased NO synthesis.^15^ However, the exact role of ADMA in tumor development is unknown. Colon cancer is highly associated with metabolic syndrome,^16^ which is the common pathophysiology for metabolic disorders such as diabetes, hypertension, and cardiovascular disease. It is, therefore, of particular significance to investigate the role of ADMA in colon cancer development.

To investigate the potential functions of ADMA in colon cancer development, we assessed the ADMA levels in colon cancer patients and normal subjects, and studied the impact of ADMA on cell proliferation and apoptosis in the LoVo colon cancer cell line. Our results showed that the ADMA serum concentration was higher in colon cancer patients than normal subjects. Additionally, ADMA treatment attenuated cell death in LoVo cells induced by serum starvation (SS) and doxorubicin hydrochloride, but did not impact the viability of normal fibroblast cells. Moreover, ADMA treatment suppressed the activation of Fas (APO-1/CD95)/JNK (SAPK) (c-Jun N terminal protein kinase/stress-activated protein kinase) pathway triggered by SS and doxorubicin in LoVo cells.

## Results

### Colon cancer patients showed a significantly higher level of ADMA in serum

**Figure 1:**
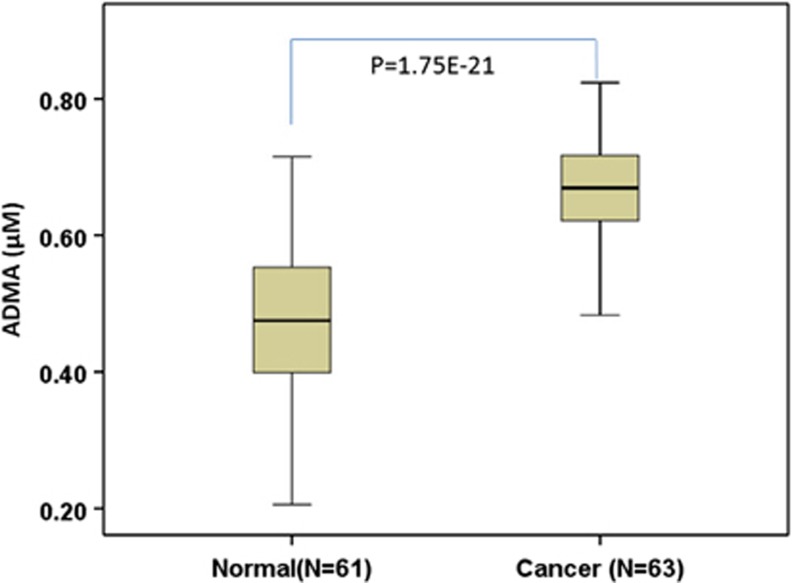
The serum ADMA levels in patients with the colon cancer and normal subjects. Serum samples were collected from 63 patients with colon cancer before surgery and 61 normal subjects. The ADMA levels were measured using ELISA. Data are presented as mean±S.D., and the statistical significance was calculated with one-way ANOVA followed by Student–Newman–Keuls (SNK) test using SPSS 20.0 software. Normal: normal subjects; cancer: colon cancer patients

To determine whether serum ADMA levels are higher in colon cancer patients, we measured serum ADMA levels in 63 patients with colon cancer and 61 normal subjects. The serum samples of all participants were collected the morning after overnight fasting, and the serum samples of colon cancer patients were collected before surgery. The sample information of colon cancer patients and normal subjects is shown in Table 1. We found that serum ADMA levels were significantly higher in patients with colon cancer compared to normal subjects (0.663 *versus* 0.471 *μ*M, *P*=1.75E−21) (Figure 1). No statistical significance was observed between males and females, either in colon cancer patients or in normal subjects (Table 1).

### ADMA attenuated SS-induced viability reduction in LoVo cells

**Figure 2:**
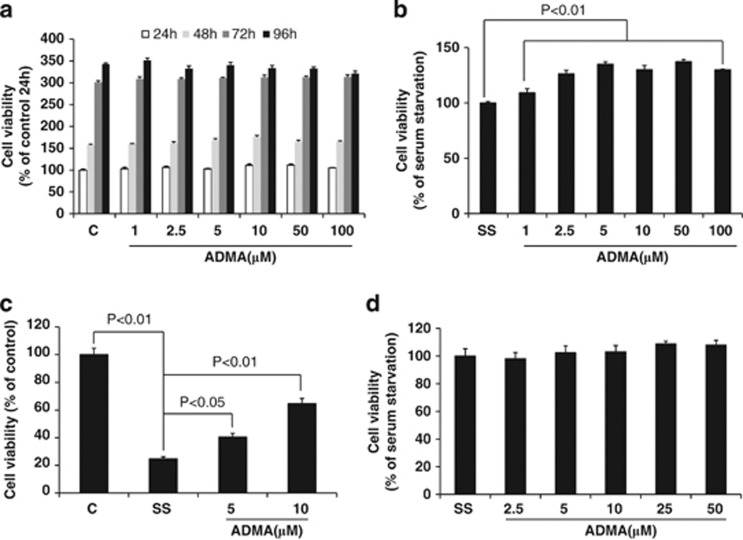
Impact of ADMA on proliferation of colon cancer cells and normal fibroblasts. LoVo cells were treated with ADMA at a series of concentrations in control (**a**) or serum-free media (**b**) for 96 h or 7 days (**c**). CRL-1459 normal fibroblasts were cultured in serum-free media for 96 h in the presence or absence of ADMA (**d**). Cell viability was measured with the CCK8 kit after specific treatments. Data are presented as mean±S.E., which are representative of at least three independent experiments. The statistical significance was calculated with student *t*-test. C: control media (10% FBS DMEM); SS: serum starvation

To determine the potential role of ADMA in colon cancer development, we first investigated the impact of ADMA on the proliferation of the LoVo colon cancer cell line. We found that ADMA treatment did not impact the proliferation rate of LoVo cells in 10% FBS Dulbecco's modified Eagle's medium (DMEM) within 96 h (Figure 2a). Since tumor cells usually experience nutrient deprivation, we examined whether ADMA could enhance the endurance of tumor cells to SS. We next cultured cells in serum-free DMEM in the presence or absence of ADMA for 96 h, and found that the cells showed higher viability in the presence of ADMA at concentrations ranging from 2.5 to 100 *μ*M (*P*<0.01, Figure 2b).To further determine the impact of ADMA on cell viability under SS conditions, we extended the SS conditions to 7 days and observed a significant reduction in cell viability compared to normal culture media (*P*<0.01). The addition of 5 or 10 *μ*M ADMA to the serum-starved cultures significantly attenuated the SS-mediated decrease of cell viability (Figure 2c). Since tumor cells are more sensitive to nutrient deprivation, we therefore tested whether ADMA could impact the viability of normal cells. We compared the viability of normal CRL-1459 fibroblast cells treated with or without ADMA for 96 h. No significant difference was observed between the two groups (Figure 2d). Interestingly, the protective effect against SS-induced viability reduction was not observed in another two colon cancer cell lines, Caco-2 and SW480, but was observed in human liver carcinoma cell line HepG2 cells (Supplementary Figure 1). Taken together, our current results indicate that ADMA can only protect some cancer cells against SS-induced cell death, but not in normal fibroblasts and some other colon cancer cells.

### ADMA inhibited SS-induced apoptosis and Fas/JNK pathway in LoVo cells

**Figure 3:**
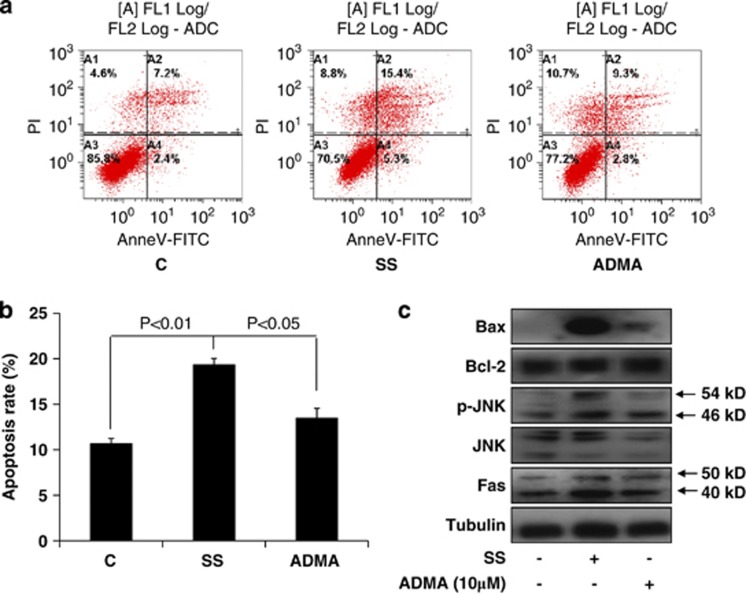
ADMA inhibited serum starvation-induced apoptosis and the Fas/JNK pathway in LoVo cells. LoVo cells were cultured in control or serum-free media in the presence or absence of 10 *μ*M ADMA for 96 h. (**a**) The apoptosis rate was assayed using flow cytometry after staining with Annexin V-FITC and propidium iodide. (**b**) Statistical results of apoptosis in different cell groups. (**c**) Western blot analysis of Fas/JNK pathway proteins as indicated. Data are presented as mean±S.E., and are representative of two independent experiments performed in triplicate. The statistical significance was calculated with Student's *t*-test. C: control media (10% FBS DMEM); SS: serum starvation

Tumor cells usually undergo increased apoptosis during SS;^17, 18^ therefore, we hypothesized that ADMA might inhibit SS-induced apoptosis in LoVo cells. To test this hypothesis, we measured the apoptosis rate of LoVo cells treated with or without 10 *μ*M ADMA for 96 h in serum-free media using flow cytometry. We found that the apoptosis rate was almost doubled upon SS compared to control cells cultured in 10% FBS DMEM. Furthermore, ADMA treatment significantly suppressed the apoptosis induced by SS (Figures 3a and b).

The induction of the Fas/JNK pathway is critical for the regulation of SS-triggered apoptosis.^19^ To investigate whether the Fas/JNK pathway is involved in the suppression of SS-induced apoptosis by ADMA treatment, we analyzed the expression of Fas, p-JNK, and JNK proteins, as well as Bax and Bcl-2 proteins with western blot. SS resulted in activation of Fas, JNK, and Bax proteins, and this activation was significantly suppressed by ADMA treatment (Figure 3c). However, there was no significant difference in the expression of Bcl-2 protein among the groups (Figure 3c). In addition, we found that SS did not induce the cytochrome *c* release and caspase-9 expression, but stimulated the levels of cleaved caspase-3 in LoVo cells, which was inhibited by ADMA (Supplementary Figure 2). These results suggest that, although the Fas/JNK pathway is critical for suppressing apoptosis by ADMA, the intrinsic apoptotic pathway may not be involved in this process.

### ADMA suppressed the activation of JNK triggered by anti-Fas mAb and C_2_-ceramide

**Figure 4:**
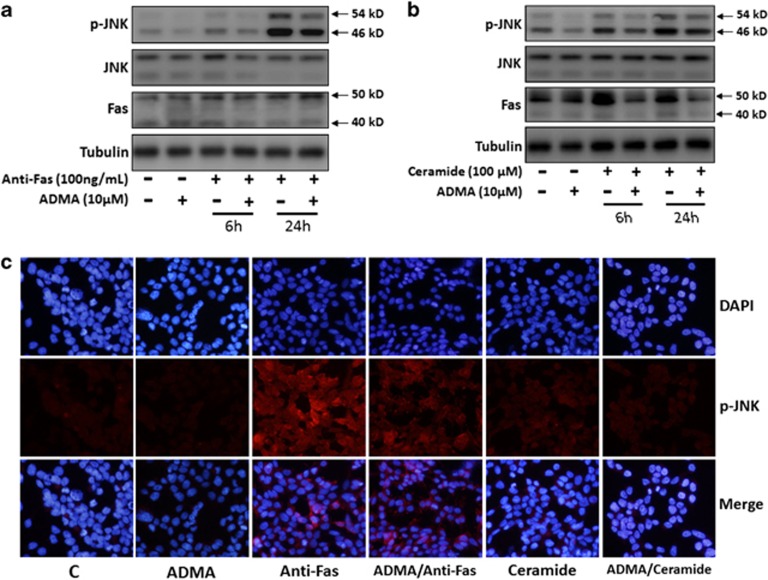
ADMA suppressed JNK activation induced by anti-Fas mAb and C_2_-ceramide in LoVo cells. LoVo cells were pretreated with 10 *μ*M ADMA for 3 days, and media was changed to fresh media containing (**a**) 100 ng/ml anti-Fas mAb or (**b**) 100 *μ*M C_2_-ceramide in the presence or absence of 10 *μ*M ADMA for an additional 6 or 24 h. Western blot analysis was performed for p-JNK, JNK, Fas, and Tubulin expression. (**c**) Immunofluorescence imaging showed JNK activation induced by 24 h treatment of anti-Fas mAb or C_2_-ceramide, and suppressed by ADMA pretreatment. Red, phosphorylated JNK; blue, 4′–6-diamidino-2-phenylindole (DAPI), nuclei. (Magnification, × 400). C: control sample

JNK is activated by anti-Fas mAb in Jurkatcells.^20^ Both SS and Fas activation are recognized as potent inducers of endogenous ceramide. The increased ceramide serves as a second messenger to activate JNK in stressful conditions.^21, 22^ To further characterize the role of ADMA in the Fas/JNK pathway, we tested whether ADMA pretreatment could prevent the activation of JNK by anti-Fas mAb and exogenous ceramide in LoVo cells. LoVo cells were pretreated with ADMA for 72 h and then treated with either 100 ng/ml anti-Fas mAb or 100 *μ*M C_2_-ceramide for 6 and 24 h. Anti-Fas mAb treatment resulted in significant activation of JNK at 24 h, which was partially suppressed by ADMA pretreatment (Figure 4a). However, anti-Fas mAb treatment did not alter the expression of Fas protein (Figure 4a). C_2_-ceramide treatment induced strong and sustained activation of Fas and JNK from 6 to 24 h, and this activation was significantly blocked by ADMA pretreatment (Figure 4b). Immunofluorescence imaging of p-JNK further confirmed these results by showing that the activation of JNK triggered by either anti-Fas mAb or exogenous ceramide treatment was suppressed by ADMA pretreatment (Figure 4c).

### ADMA attenuated doxorubicin-induced cell death and Fas/JNK pathway activation

**Figure 5:**
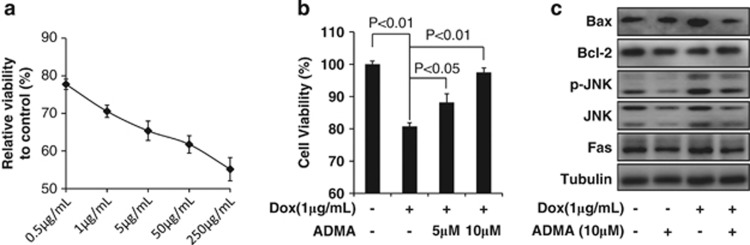
ADMA attenuated doxorubicin-triggered cell death and activation of Fas/JNK pathway. (**a**) LoVo cells were treated with doxorubicin for 24 h, and cell viability was measured with CCK-8. (**b**) LoVo cells were pretreated with 10 *μ*M ADMA for 3 days, and media was changed to fresh media containing 1 *μ*g/ml doxorubicin for 24 h. Cell viability was measured with CCK-8. (**c**) Western blot analysis of Fas/JNK pathway proteins as indicated. Data are presented as mean±S.E., and are representative of three independent experiments. The statistical significance was calculated with Student *t*-test. Dox: doxorubicin

Given the protective effect of ADMA against SS-induced apoptosis, we postulated that ADMA might impair the efficacy of antitumor drugs. LoVo cells are sensitive to doxorubicin,^23^ a widely used chemotherapy agent in the clinic. To test our hypothesis, we first treated LoVo cells with doxorubicin at a series of concentrations (0.5–250 *μ*g/ml), and confirmed an apparent dose-dependent cell death (Figure 5a). Next, LoVo cells were pretreated with or without ADMA for 3 days, and the media was changed to one containing 1 *μ*g/ml doxorubicin for 24 h. We found that pretreatment of 5 or 10 *μ*M ADMA protected LoVo cells from doxorubicin-induced cell death (Figure 5b).

To further investigate whether the attenuation of doxorubicin-induced cell death by ADMA is due to suppression of the Fas/JNK pathway, we analyzed the expression levels of Fas, p-JNK, JNK, Bax, and Bcl-2 proteins. Our results showed that the expression of Fas, p-JNK, and Bax was significantly increased by doxorubicin treatment, while ADMA pretreatment suppressed the activation of these proteins (Figure 5c). Together, these results suggest that ADMA can attenuate the antitumor effect of doxorubicin by suppressing the Fas/JNK pathway in LoVo cells.

## Discussion

Our results revealed that (1) serum ADMA concentration was elevated in patients with colon cancer; (2) ADMA treatment attenuated SS-induced apoptosis in LoVo cells via suppression of the Fas/JNK pathway; and (3) ADMA ameliorated doxorubicin-induced cell death and inhibited activation of Fas and JNK in LoVo cells.

It should be noted that the elevated serum ADMA levels in patients with colon cancer may be confounded by comorbidities of metabolic disorders in the cohort, although their average BMI value (Table 1) is not greater than that of the control subjects. ADMA is an endogenous inhibitor of nitric oxide synthase, which is elevated in cardiovascular diseases and associated with endothelial dysfunction.^24^ ADMA has been reported to induce apoptosis in human umbilical vein endothelial cells (HUVECs),^25^ and elicit significant changes in coronary artery endothelial cell gene expression.^12^ ADMA is also a structural analog of metformin, and they have opposite functions in multiple metabolic and signaling pathways that coordinate energy metabolism, cell growth, and molecule synthesis.^26^ Metformin treatment was reported to lower ADMA levels in patients with type 2 diabetes.^27^ Given the preventive effect of metformin against various cancers,^28, 29, 30, 31^ ADMA may also play important roles in cancer development. A recent study revealed the dysfunction of protein arginine methyltransferases in various types of human cancers, leading to the elevation of ADMA levels in patients with various cancers.^13^ In the current study, we demonstrated that serum ADMA levels are higher in colon cancer patients than in normal subjects.

A previous report showed that either 2 or 100 *μ*M ADMA treatment did not alter the viability of HUVECs.^12^ In the present study, we found that ADMA had no impact on the viability of LoVo cells in normal culture media containing 10% FBS. However, we observed that ADMA treatment significantly reduced the viability of LoVo cells in serum-free DMEM, but not in another two colon cancer cells: Caco-2 and SW480 cells. These results are beyond our expectation; however, we speculate that the biological function of ADMA in cell lines or human body may be influenced by the genetic background or other confounding factors. SS usually triggers apoptosis in tumor cells,^17, 32^ and the activation of the anti-apoptosis signaling pathway is critical for tumorigenesis and development of drug resistance in cancer cells. ADMA has been recognized as an independent risk factor for cardiovascular disease,^33^ and an inducer of apoptosis in endothelial cells.^25^ However, it also antagonizes glutamate-induced cytotoxicity and apoptosis by upregulation of Bcl-2 in PC12 cells.^34^ These results suggest that the function of ADMA in apoptosis is cell type-specific.

The mechanisms underlying SS-induced apoptosis are complicated and vary across different cell lines and under different starvation conditions.^35^ Fas is an important member of tumor necrosis factor receptor superfamily, and stimulation of Fas by its natural ligand (FasL) leads to apoptosis in response to various stresses.^36^ Nevertheless, we did not observe an increase in FasL expression upon SS (data not shown), which is consistent with the report on type II Jurkat cells.^19^ We observed that ADMA suppressed the expression of Fas protein induced by SS in LoVo cells. However, a similar effect was not observed in cell surface Fas level detected by flow cytometry (Supplementary Figure 3). We are not quite clear about the exact reasons for the current inconsistent results; however, it is speculated that the inconsistency may be associated with the differences in cell passages between these two measurements because of the fact that cell passage numbers are important factors for influencing alteration in cell line physiology and response to stimuli.^37, 38^ Activation of JNK is critical for mediating apoptosis triggered by Fas.^39^ Activated JNK transmits apoptotic signals to the mitochondrial apoptosis-related Bcl-2 family proteins,^40, 41^ which include anti-apoptotic proteins (e.g. Bcl-2, Bcl-xL) and pro-apoptotic proteins (e.g. Bax, Bad). SS-induced Bax promotes apoptosis by competing with Bcl-2 protein, while the presence of Bcl-2 inhibits the activation of Bax following the death signal.^42, 43^ Our results showed that SS activated JNK and Bax, whereas ADMA treatment antagonized SS-induced activation of JNK and Bax in LoVo cells. However, the expression of Bcl-2 protein was not altered either by SS or by ADMA treatment. The members of Bcl-2 family are crucial for regulating the apoptotic pathway, which are subdivided into prodeath and antideath members.^44^ The prodeath members, Bax and Bak, usually stimulate cytochrome *c* release by forming oligomerization, which triggers the intrinsic apoptotic pathway.^45^ Activated JNK is pro-apoptotic by stimulating the prodeath member of Bcl-2 family, that is, Bax.^46^ In our current report, we found that cytochrome *c* release and caspase-9 expression were not induced by SS, in spite of the activation of Fas/JNK and Bax by SS. However, the cleaved caspase-3 fragments were increased by SS, but reduced by ADMA treatment. These results suggest that ADMA may antagonize SS-induced apoptosis through suppression of the Fas/JNK pathway; however, the mechanism acting between Fas/JNK activation and the effector caspase, caspase-3, needs further investigation in our model.

Ceramide is usually formed under conditions of stress, such as SS, UV irradiation, chemotherapeutic drugs, and oxidative stress.^21, 47, 48^ SS is recognized as the strongest inducer of intracellular ceramide generation,^49^ which precedes the activation of JNK.^21^ The activation of JNK after SS or exogenous ceramide treatment can only be detected in wild-type Jurkat cells, but not in FasL-resistant Jurkat cell clones,^19^ indicating that JNK activation in response to these stresses is Fas-dependent. On the other hand, Fas can also trigger the generation of ceramide. Although the regulation between Fas and ceramide is complicated,^50, 51^ the activation of JNK is the common pathway in mediating Fas and ceramide-induced apoptosis.^19^ In the current study, we observed that ADMA pretreatment antagonized the activation of Fas and JNK triggered by ceramide, and JNK activation by anti-Fas mAbin LoVo cells.The blockage of anti-Fas mAb and C_2_-ceramide-induced JNK activation by ADMA pretreatment confirms the suppression of the Fas/JNK pathway by ADMA treatment in LoVo cells. Nevertheless, previous reports have also shown that ADMA can induce the expression of p-JNK, glucose-regulated protein 78, and trigger endoplasmic reticulum stress in 3T3-L1 adipocytes,^52^ as well as apoptosis via activation of p38 mitogen-activated protein kinases in HUVECs.^25^ The discrepant functions of ADMA in apoptosis between our current study and previous reports suggest that ADMA may play different roles in different cell lines and stresses.

Tumor cells usually downregulate Fas expression to acquire an apoptosis-resistant phenotype, which is a hallmark of metastatic human colorectal cancer. Epigenetic inhibitors decitabine and vorinostat cooperate to upregulate Fas expression in metastatic human colon carcinoma cells, leading to sensitization to FasL-induced apoptosis.^53^ Doxorubicin is effective in the treatment of a broad range of solid human malignancies in the clinic by activating Fas signaling.^54^ Moreover, the combination of doxorubicin with death receptor antibody exhibits synergistic induction of cell death through activation of the JNK/p38 pathway.^55^ In our study, we observed that ADMA pretreatment could protect LoVo cells from doxorubicin-induced cell death, but not 5-fluorouracil (5-FU) (Supplementary Figure 4). Further analysis showed that the Fas/JNK pathway was stimulated by doxorubicin, but not by 5-FU, which may account for the different effects of ADMA in doxorubicin and 5-FU therapy. Although 5-FU has been reported to induce apoptosis via the Fas pathway in liver metastases of colorectal cancer patients,^56^ and stimulate p-JNK in colorectal cancer cells,^57^ our current results suggest the probable existence of a Fas/JNK-independent mechanism in chemotherapy of 5-FU, as well as the importance of Fas/JNK pathway in protection against apoptosis by ADMA.

In conclusion, we show in this report that serum ADMA levels are elevated in colon cancer patients. ADMA attenuates SS and doxorubicin-induced apoptosis in LoVo cells via a mechanism of suppressing the Fas/JNK pathway. However, the cell type-specific anti-apoptosis effect of ADMA in different cancer cells suggests that the biological functions of ADMA may vary in the context of diversified genetic background. Further investigation is needed to determine the role of ADMA in other cancer cell lines and colon cancer animal models.

## Materials and Methods

### Reagents and cell culture

Doxorubicin hydrochloride, 5-FU, C_2_-ceramide and ADMA were purchased from Sigma-Aldrich (St Louis, MO, USA). Anti-Fas mAb (human, activating) clone CH11 (Catalog Number: 05–201) was purchased from EMD Millipore Corporation (Billerica, MA, USA). DMEM was purchased from Life Technology (Grand Island, NY, USA), and FBS was purchased from Atlanta Biologicals (Lawrenceville, GA, USA). The cell counting kit-8 was purchased from Enzo Life Sciences (Farmingdale, NY, USA). Human colon cancer LoVo cells (CCL-229), Caco-2 (HTB-37), SW480 (CCL-228), normal fibroblasts (CRL-1459), and human liver cancer cells HepG2 (HB-8065) from ATCC were routinely cultured in 10-cm dishes at 37 °C in a humidified atmosphere of 5% CO_2_ in 10% FBS DMEM supplemented with 100 U/ml penicillin and 100 *μ*g/ml streptomycin.

### Measurement of ADMA concentration with the ELISA

The serum ADMA levels were measured using the ELISA method (Catalog Number: AAP31-K02, Eagle Biosciences, Inc., Boston, MA, USA), according to the manufacturer's instructions. The detection limit of the ADMA ELISA kit is 0.05 *μ*M.

### Sample information

Serum samples were collected from 63 patients with colon cancer before surgery and 61 normal subjects at Fudan University Shanghai Cancer Center, Shanghai, China. All participants signed informed consent before they were included into the study. The study was approved by the Institutional Review Board of Fudan University. The sample information is shown in Table 1.

### Determination of cell viability

Cells were seeded in 96-well plates at 2 × 10^4^ cells/well in 10% FBS DMEM. After 24-h culture, cells were treated with or without ADMA in 10% FBS DMEM or serum-free DMEM at ranging concentrations, from 1 to 100 *μ*M, for the specified number of hours. Then, cell viability was determined using the cell counting kit-8, according to the instructions.

### Measurement of apoptosis with flow cytometry

After LoVo cells were treated with or without 10 *μ*M ADMA for 96 h in serum-free medium, the cells were digested with 0.25% trypsin and 5 × 10^5^ cells in each sample group were collected. The collected cells were resuspended in 500 *μ*l binding buffer, and 5 *μ*l of Annexin V-FITC and propidium iodide (ab14085, Abcam, Cambridge, MA, USA) was added. The cells were incubated at room temperature for 5 min in the dark, and apoptosis was measured using flow cytometry (Beckman Coulter, Indianapolis, IN, USA).

### Western blotting

LoVo cells were treated under specific conditions in six-well plates and the total protein was extracted using the Mammalian Protein Extraction reagent (Thermo Scientific, Rockford, IL, USA). The protein concentrations were determined with the Pierce Coomassie Protein Assay Kit (Thermo Scientific). A total of 5 *μ*g of protein was loaded into each lane and separated by 12% SDS-PAGE, and then transferred to a PVDF membrane (BIO-RAD, Hercules, CA, USA). The membrane was blocked with 5% bovine serum albumin in TBST (20 mM Tris-HCl, 137 mM NaCl, and 0.1% Tween 20, pH 7.5) at room temperature. The membranes were incubated with primary antibodies (1 : 1000 dilution) overnight at 4 °C. Following incubation with HRP-conjugated secondary antibodies, the membranes were exposed using a Fujifilm LAS 4000 image system. The bands were quantified by optical density ratio using *α*-tubulin as a control. The primary antibodies used for analysis were Bax, Bcl-2, Fas, p-JNK, and JNK; all antibodies were purchased from Cell Signaling Technology (Danvers, MA, USA).

### Immunofluorescence staining

After Anti-Fas mAb or C_2_-ceramide treatment, LoVo cells were fixed in 4% paraformaldehyde for 20 min and permeabilized in 0.2% Triton X-100 for 10 min at 4 °C. After three washes with PBS, plates were treated with blocking buffer containing 10% goat serum and 5% bovine serum albumin in PBS for 1 h. Mouse anti-p-JNK (Thr^183-^Tyr^185^JNK, 1 : 100, Cell Signaling Technology) was added in PBS containing 1% bovine serum albumin and plates were incubated overnight at 4 °C. Plates were rinsed with PBS and incubated with an anti-mouse IgG (H+L), F(ab')_2_ Fragment (Alexa Fluor 594 Conjugate, 1 : 1000, Cell Signaling Technology) for 1 h. After counterstaining with 300 nM 4′-6-diamidino-2-phenylindole (DAPI) (Cell Signaling Technology), cells were mounted under coverslips using a drop of AQUA-POLY/MOUNT (Polysciences, Inc., Warrington, PA, USA). Images were acquired with an inverted fluorescence microscope IX71 (Olympus, Center Valley, PA, USA).

### Statistical analysis

Data are expressed as means±S.E.M., except for as indicated. Significant differences were assessed either by Student's *t*-test or one-way ANOVA followed by the Student–Newman–Keuls (SNK) test. *P*<0.05 was considered statistically significant.

## Figures and Tables

**Table 1:** Clinical information of colon cancer patients and normal subjects

**Subjects**	**Normal subjects**	**Colon cancer patients**
*Serum ADMA (*μ*M)*		
Total	0.471±0.013	0.663±0.011*
Male	0.474±0.024	0.650±0.016*
Female	0.469±0.016	0.669±0.014*
		
*Number of patients (total)*	61	63
Male	17	26
Female	44	37
		
*Age (average)*		
Male	58	65
Female	55	57
		
*BMI (average)*		
Male	25	24
Female	26	23

Abbreviation: BMI, body mass index

Serum ADMA level was measured with ELISA. Data are expressed as mean±S.E. Significant differences were assessed by one-way ANOVA followed by the Student–Newman–Keuls (SNK) test. *P<0.01 compared to normal subjects

## Abbreviations

JNK/SAPK: c-Jun N terminal protein kinase/stress-activated protein kinase
SS: serum starvation
FasL: Fas ligand
ADMA: asymmetric dimethylarginine
5-FU: 5-fluorouracil
