# Supplementary figures and images for: Asymmetric dimethylarginine attenuates serum starvation-induced apoptosis via suppression of the Fas (APO-1/CD95)/JNK (SAPK) pathway
Source: Cell Death Dis. 2013 Oct 3;4(10):e830–. doi: 10.1038/cddis.2013.345 (PMC3824655; doi:10.1038/cddis.2013.345)

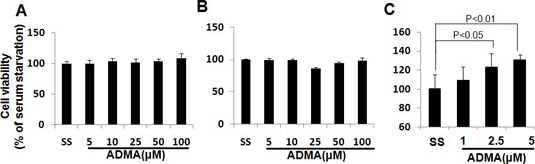

Supplement: Supplementary Figure 1 [file cddis2013345x1.tif]

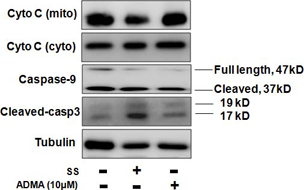

Supplement: Supplementary Figure 2 [file cddis2013345x2.tif]

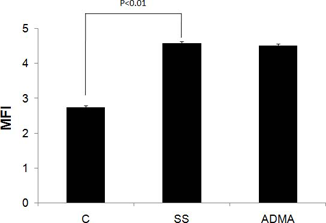

Supplement: Supplementary Figure 3 [file cddis2013345x3.tif]

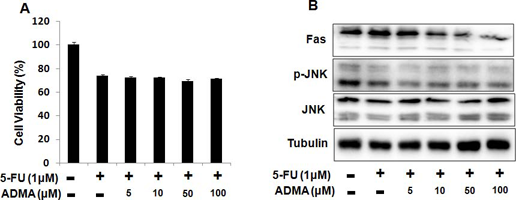

Supplement: Supplementary Figure 4 [file cddis2013345x4.tif]
