# Supplementary material for: Asymmetric dimethylarginine attenuates serum starvation-induced apoptosis via suppression of the Fas (APO-1/CD95)/JNK (SAPK) pathway
Source: Cell Death Dis. 2013 Oct 3;4(10):e830–. doi: 10.1038/cddis.2013.345 (PMC3824655; doi:10.1038/cddis.2013.345)
Supplement: Supplementary Information [file cddis2013345x5.doc]

**Supplementary Information**

**Asymmetric dimethylarginineattenuates serum starvation-inducedapoptosis via suppression of Fas (APO-1/CD95)/JNK(SAPK) pathway**

Houkai Li, Yalu Zhou, Aihua Zhao, YunpingQiu, GuoxiangXie, Qi Jiang, XiaojiaoZheng, Wei Zhong, Xiuhua Sun, Zhanxiang Zhou, Wei Jia

**Material and Methods**

**Analysis of cell surface Fas level with flow cytometry**

The analysis of cell surface Fas level was performed according to method with minor modifications [^1^](#_ENREF_1). Briefly, after LoVo cells were treated with or without 10 μM ADMA for 96 h in serum-free medium, the cells were digested with 0.25% trypsin and resuspended in 2% FBS DMEM at the concentration of 5× 10^6^ cells / mL. Then, the cells of each sample were subdivided into two tubes with 5 × 10^5^ cells per tube. A 20μl of anti-Fas mAb, Clone CH11 (Cat no. 05-201, EMD Millipore, US) or 20μl of mouse IgM negative control, clone GC323 (Cat no. MABC008, EMD Millipore, US) was added to each tube and incubated for 30 min in the dark at room temperature. Then, the cells were washed with 2% FBS DMEM twice and incubated with FITC-conjugated Goat anti-mouse IgM (Cat no. AP500F, EMD Millipore, US) ) for 30 min in the dark at room temperature. Finally, suspended the cells with 500μl 2% FBS DMEM, and analyzed with flow cytometry (Beckman Coulter, Indianapolis, IN, USA). The mean fluorescence intensity (MFI) of each group was analyzed using a CXP program (Beckman Coulter).

**Supplementary Figures 1-4**

**Supplementary Figure 1. Impact of ADMA on cell viability in serum starvation condition.** Caco2 (**A**), SW480 (**B**) and HepG2 (**C**) cells were treated with ADMA at indicated concentrations in serum-free DMEM for 96h. Then, cell viability was measured with the CCK8 kit. Data are presented as mean ± S.E., which are representative of two independent experiments. The statistical significance was calculated with student *t*-test. SS: serum starvation.

**Supplementary Figure 2. Impacts of ADMA on expression of caspases and cytochrome C.** LoVo cells were treated with or without 10 μM ADMA in serum-free media for 96h. Then, the mitochondrial and cytoplasmic protein was collected with method described in Material and Methods. Expression of cleaved caspase-3, caspase-9 and cytochrome C was analyzed with western blot. SS: serum starvation.

**Supplementary Figure 3. Impacts of ADMA on cell surface Fas protein levels.** LoVo cells were treated with or without 10 μM ADMA in serum-free media for 96h. Then, cells were collected and a 20μl of anti-Fas mAb, Clone CH11 or 20μl of mouse IgM negative control, clone GC323 was added to each tube and incubated for 30 min in the dark at room temperature. Then, the cells were washed with 2% FBS DMEM twice and incubated with FITC-conjugated Goat anti-mouse IgM for 30 min in the dark at room temperature, and analyzed with flow cytometry. The mean fluorescence intensity (MFI) was calculated. Data are presented as mean ± S.D., which are representative of two independent experiments. The statistical significance was calculated with student *t*-test. SS: serum starvation.

**Supplementary Figure 4. ADMA pretreatment did not attenuate 5-FU-induced cell death.** LoVo cells were pretreated with ADMA at indicated concentrations for 3 days, and then, media was changed to fresh media containing 1μM 5-FU for 24h. Cell viability was measured with CCK-8 (**A**). (**B**) Western blot analysis of Fas/JNK pathway proteins as indicated.

1. Moller, P., K. Koretz, F. Leithauser, S. Bruderlein, C. Henne, A. Quentmeier, et al., Expression of APO-1 (CD95), a member of the NGF/TNF receptor superfamily, in normal and neoplastic colon epithelium*.* *Int J Cancer*. 1994. **57**:371-377.
